# Intake of wholegrain products and risk of colorectal cancers in the Diet, Cancer and Health cohort study

**DOI:** 10.1038/sj.bjc.6605806

**Published:** 2010-08-24

**Authors:** R Egeberg, A Olsen, S Loft, J Christensen, N F Johnsen, K Overvad, A Tjønneland

**Affiliations:** 1 Institute of Cancer Epidemiology, The Danish Cancer Society, Strandboulevarden 49, DK-2100 Copenhagen Ø, Denmark; 2 Institute of Public Health, University of Copenhagen, Øster Farimagsgade 5, PO box 2099, DK-1014 Copenhagen K, Denmark; 3 Department of Epidemiology, School of Public Health, Aarhus University, Bartholins Allé 2, DK-8000 Aarhus C, Denmark; 4 Department of Cardiology, Aalborg Hospital, Aarhus University Hospital, Sdr. Skovvej 15, DK-9000 Aalborg, Denmark

**Keywords:** wholegrain products, colon cancer, rectal cancer, prospective study

## Abstract

**Background:**

Consumption of wholegrain (WG) products may protect against colon and rectal cancer.

**Methods:**

The associations between total and individual WG product consumption and colon and rectal cancer risk were prospectively examined using data on 461 incident cases of colon cancer and 283 incident cases of rectal cancer that developed during 10.6 years (median) of follow-up among 26 630 men and 29 189 women taking part in the Diet, Cancer and Health cohort. Incidence rate ratios (IRRs) of colon and rectal cancer related to total or individual WG product intake were calculated using Cox regression.

**Results:**

Higher WG product intake was associated with lower risk of colon cancer and rectal cancer in men. The adjusted IRR (95% CI) was 0.85 (0.77–0.94) for colon cancer and 0.90 (0.80–1.01) for rectal cancer per daily 50 g increment in intake. For colon cancer the association was confined to intake of WG bread in particular. No consistent associations between total or individual WG product consumption and colon or rectal cancer risk were observed in women.

**Conclusion:**

The findings suggest that higher total WG product intake is associated with a lower risk of colon and perhaps rectal cancer in men, but not in women.

Wholegrain (WG) products are widely recommended as part of a healthy diet in many countries, but their significance for cancer prevention is not understood. With respect to colon and rectal cancer, a preventable effect of WG products seem plausible as they are rich in a variety of components including dietary fibres and other bioactive substances such as vitamins (vitamins belonging to the B group and vitamin E), minerals (e.g. magnesium, selenium), antioxidants, phytooestrogens and other phytochemicals which all are anticipated to influence cancer risk through various mechanisms (Slavin, 2004).

Although much data from case–control studies are supportive of a protective association between WG product intake and colorectal cancer risk (Jacobs *et al*, 1998; Levi *et al*, 1999; La *et al*, 2003; Slattery *et al*, 2004), evidence from prospective studies is limited, including two studies showing a protective association (Larsson *et al*, 2005; Schatzkin *et al*, 2007) and two showing no overall association (Pietinen *et al*, 1999; McCullough *et al*, 2003). Few studies have focussed, as we have, on specific types of WG products, although high intake of rye-based products have been associated with a lower risk of colorectal cancer in some prospective studies (Pietinen *et al*, 1999; Larsson *et al*, 2005).

Due to the inconsistent results and limited number of prospective studies, the impact of WG product intake on the risk of colon and rectal cancer is still not clear-cut. Thus, the aim of this study was to examine the association between intake of total and individual WG products in relation to risk of colon and rectal cancer within the Danish Diet, Cancer and Health prospective cohort study. Wholegrain products, especially WG rye bread, are one of the stable foods in Denmark where intake is among the highest in the world. Because of that, the Diet, Cancer and Health study present a unique setting for a study to contribute to the further clarification of any potential role of WG products on risk of colon and rectal cancer. As dietary fibres, one of the proposed components responsible for the potential protective effect of WG products, and colorectal cancer risk has already been examined in the European Prospective Investigation into Nutrition and Cancer (EPIC) (Bingham *et al*, 2003) (of which the present cohort is part), specific fibre analyses are not included in this study.

## Subjects and methods

The Diet, Cancer and Health study is a prospective cohort study (Tjonneland *et al*, 2007). In brief, between 1993 and 1997, 160 725 Danish men and women were invited to participate, the inclusion criteria being age 50–64 years, living in the greater Copenhagen and Aarhus areas, born in Denmark and not registered with a previous cancer diagnosis in the Danish Cancer Registry (Storm *et al*, 1997). Subjects were identified by the unique ten-digit identification number, allocated to every citizen by the Danish Central Population Registry, of whom 27 178 men and 29 875 women participated. All participants attended one of two established study centres in Copenhagen and Aarhus. The Diet, Cancer and Health cohort study and the present study were approved by the Regional Ethical Committees on human studies in Copenhagen and Aarhus, and by the Danish Data Protection Agency. The present study is based on data from 26 630 male participants and 29 189 female participants after exclusion of subjects with a previous cancer diagnosis before baseline (*n*=571) and subjects who did not fill in the lifestyle questionnaire (*n*=37). In addition, we further excluded 285 men and 341 women because of missing information on variables considered in the analyses.

Diet was measured by a 192-item food frequency questionnaire (FFQ) that each cohort participants received by mail before their visit to one of the two study centres. A description of the development and validation of this FFQ has been published previously (Overvad *et al*, 1991; Tjonneland *et al*, 1991, 1992; Haraldsdottir *et al*, 1994). The FFQ was designed specifically for the study population, aiming to capture the average intake of different food and beverage items over the past 12 months before study inclusion. Dietary consumption was assessed in 12 categories of predefined responses, ranging from ‘never’ to ‘8 times or more per day’. Daily intakes of foods and nutrients were calculated for each participant by the software programme FoodCalc (www.ibt.ku.dk/jesper/foodcalc/) using population-specific standardised recipes and portion sizes. We considered total daily intake (g per day) of WG products as the main exposure variable. This food group consisted of intake of WG rye bread, WG bread and oatmeal. Secondary analyses were conducted for the three individual WG products (WG rye bread, WG bread and oatmeal).

During the study centre visit, participants also completed a lifestyle questionnaire including questions regarding social factors, health status, reproductive factors and lifestyle habits. From this questionnaire the following information was available: years of school education, use of hormone replacement therapy (HRT) and leisure time physical activity. The FFQ and the lifestyle questionnaire were both processed during the visit by optical scanning and checked so that missing or unclear information could be clarified with the participant before he/she left the study clinic. A few missing values were accepted in the lifestyle questionnaire, but not in the FFQ. Furthermore, participants had their anthropometrical measurements (e.g., height and weight) recorded by trained health professionals at the study clinics. Body mass index (BMI) was calculated as weight (kg) per height^2^ (m^2^).

All cohort members were linked to the Central Population Registry for information on vital status and emigration. Each cohort member was followed up for colon or rectal cancer from the date of entry, that is, date of visit to the study centre until the date of diagnosis of any cancer (except non-melanoma skin cancer), the date of death, date of emigration or 27 April 2006. Information on cancer incidence was obtained by linkage to the Danish Cancer Registry using the personal identification number of each participant. All individuals in Denmark who are diagnosed with cancer are registered herein using the Central Population Registry system (Storm *et al*, 1997). Definition of colon and rectal cancer was based on the tenth Revision of the International Classification of Diseases. Cancers of the colon included tumours occurring at the colon (C18.0–C18.9 and C19). Proximal colon tumours included the caecum, appendix, ascending colon, hepatic flexure, transverse colon and splenic flexure (C18.1–C18.5). Distal colon tumours included the descending (C18.6) and sigmoid (C18.7) colon and tumours occurring at the rectosigmoid junction (C19.9). Colon tumours at overlapping lesions (C18.8) and colon NOS (C18.9) were grouped among all colon cancers only. Rectal cancers included tumours occurring at the rectum (C20). Anal canal tumours were not considered in this analysis.

### Statistical analysis

The analyses of the relation between the WG product variables and colon or rectal cancer rates were based on the Cox proportional hazard model (including time-dependent variables) using age as the time axis to ensure that the estimation procedure was based on comparisons of individuals at the same age, which allowed for age adjustment to prevent confounding by age. The other time variable, time under study, was included as a time-dependent variable modelled by a linear spline with a boundary at 1, 2 and 3 years after entry into the study cohort to allow for a possible ‘healthy-participants’ effect. A linear spline was used because this allows a steady increase in the rate during the first year of follow-up (Greenland, 1995b). Tests of effect modification by sex were performed using the likelihood ratio test. Test for interaction between sex and total WG product intake revealed a significant interaction for both colon (*P*=0.0496) and rectal cancer (*P*=0.0008). Consequently, data were analysed separately by sex.

All models were adjusted for baseline values of potential colon or rectal cancer risk factors including BMI (weight (kg) per height (m)^2^; *continuous*), alcohol intake (g per day; *continuous*), school education (low (⩽7 years), medium (8–10 years) and high (⩾11 years)), intake of red and processed meat (g per day; *continuous*) and use of HRT in women only (never, past or current use). Furthermore, colon cancer models were adjusted for leisure time physical activity (includes the six different activities: walking, cycling, sports, housework, gardening and hobby work; the number of activities with participation is defined as >0 h per week of activity; *continuous*) (Johnsen *et al*, 2006).

All quantitative variables were entered linearly in the Cox model because this is biologically more reasonable than the step functions corresponding to categorisation and, furthermore, it increases the power of the analyses (Greenland, 1995a). The linearity of the associations was evaluated graphically by linear splines with three boundaries placed at the quartiles among cases. As we found no significant departures from linearity, all quantitative variables were entered linearly in the model (Greenland, 1995b).

In the analyses, WG product variables included intakes in grams per day of total WG products and individual WG products (WG rye bread, WG bread and oatmeal). The incidence rate ratios (IRRs) of the associations of linear WG product variables were presented as the IRRs associated with a higher intake of 25 g per day (individual WG products) or 50 g per day (total WG products) based on an evaluation of the inter-quartile range among cases (men and women, colon and rectal cancer cases combined). The continuous analyses were further supplemented with categorical analyses. In these categorical analyses, the WG product variables were categorised into quartiles based on the distribution among cases (men and women, colon and rectal cancer cases combined).

Two-sided 95% confidence intervals (CIs) for the IRRs were calculated based on Wald's test of the Cox regression parameter, that is, on the log rate ratio scale. The procedure PHREG in SAS (release 9.1; SAS Institute, Cary, NC, USA) on a TextPad platform was used for the statistical analyses.

## Results

During a median follow-up of 10.6 years, the distributions of diagnosed cancer cases were as follows: 244 colon cancers (89 proximal, 140 distal and 15 unknown) and 169 rectal cancers in men, and 217 colon cancers (84 proximal, 118 distal and 15 unknown) and 114 rectal cancers in women. Baseline characteristics of the total cohort and cancer cases presented separately for men and women are given in Table 1. Compared with the total cohort of men, male colon and rectal cancer cases had a higher intake of alcohol and red and processed meat and were less educated. Female colon and rectal cancer cases had a lower alcohol intake, were less educated and more likely to be never HRT users compared with the total cohort of women. In addition, colon cancer cases had a slightly higher intake of red and processed meat compared with the total cohort of women.

Table 2 presents the IRRs (95% CI) for colon and rectal cancer according to intake of total and individual WG products in men and women. In men, higher intake of total WG products was associated with a statistically significant 15% lower incidence rate of colon cancer (IRR 0.85, 95% CI 0.77–0.94, per each increment in intake of 50 g per day) and a borderline statistically significant 10% lower incidence rate of rectal cancer (IRR 0.90, 95% CI 0.80–1.01, per each increment in intake of 50 g per day). In women, no consistent associations were observed for either colon or rectal cancer.

Among men, a borderline statistically significant lower risk of colon cancer was found for higher WG rye bread consumption (IRR 0.94, 95% CI 0.88–1.01). Higher WG bread intake was statistically significantly associated with a lower risk of colon cancer. For every increment in intake of 25 g per day, the adjusted IRR was 0.89 (95% CI 0.82–0.97). Oatmeal consumption was not associated with the risk of colon cancer among men (Table 2). No associations were found between any of the individual WG products and rectal cancer among men and colon or rectal cancer among women (Table 2).

Among men, intake of total WG products was associated with a lower risk of both proximal and distal colon cancer (IRR_proximal colon_ 0.78, 95% CI 0.66–0.92 per each increment in intake of 50 g per day; IRR_distal colon_ 0.90, 95% CI 0.79–1.02 per each increment in intake of 50 g per day). Higher intakes of WG rye bread (IRR 0.89, 95% CI 0.79–1.01 per each increment in intake of 25 g per day) and WG bread (IRR 0.88, 95% CI 0.76–1.01 per each increment in intake of 25 g per day), but not oatmeal, were borderline statistically significantly associated with lower risk of proximal colon cancer. Only a higher intake of WG bread was associated with a lower risk of distal colon cancer among men (IRR 0.89, 95% CI 0.80–1.00 per each increment in intake of 25 g per day). In women, no associations were observed between intake of total or individual WG products and risk of either proximal or distal colon cancer (data not shown).

## Discussion

In this population-based prospective cohort of Danish men and women, we found that higher intake of total WG products was associated with a significant 15% lower risk of colon cancer, and also a tendency towards lower risk of rectal cancer among men. The association with colon cancer risk among men seemed to be confined to intake of WG bread in particular. We observed no associations of risk of colon or rectal cancer with either total consumption of WG products or with individual types of WG products among women.

The strengths of this study include its prospective design and high follow-up rate, which eliminates potential recall bias or selection bias; and complete and valid identification of all cancer cases through linkage to the Danish Cancer Registry. Moreover, comprehensive information was also available on potential confounders. Our study also had several potential limitations. First, the relatively small number of both colon and rectal cancer cases, although with more than 10 years of follow-up (median), remains a limitation. This is mainly true for colon cancer subsite-specific analyses and rectal cancer analyses for each sex. Second, although our dietary assessment method is validated (Tjonneland *et al*, 1991, 1992; Haraldsdottir *et al*, 1994), some measurement error in the assessment of the dietary intake is inevitable. The use of a self-administered FFQ that, in addition, was not specifically developed to estimate WG product intake among our study population may have contributed to misclassification. This misclassification because of the prospective design of the study is probably non-differential and may have caused an attenuation of results. Finally, although control for potential colon and rectal cancer risk factors in the analyses did not substantially change the estimates, as residual confounding from these variables cannot fully explain the study findings, we cannot exclude the possibility that the study results have been biased by confounding by unaccounted factors.

Four cohort studies have explored associations between total WG product intake and risk of colorectal cancer or its subsites (Pietinen *et al*, 1999; McCullough *et al*, 2003; Larsson *et al*, 2005; Schatzkin *et al*, 2007). Similar to our findings for men, Schatzkin *et al* (2007) found in the NIH-AARP Diet and Health study a significant lower risk of colorectal cancer among men for a higher WG intake. In contrast, in two other cohort studies no associations were shown (Pietinen *et al*, 1999; McCullough *et al*, 2003). Quite unexpected, intake of total WG products was not associated with risk of either colon or rectal cancer among women in our study. Although these findings agree with results from one previous cohort study (McCullough *et al*, 2003), they disagree with results from two other recent larger cohort studies presenting results separately for women (Larsson *et al*, 2005; Schatzkin *et al*, 2007). In the Swedish Mammography cohort, Larsson *et al* (2005) reported a statistically significant lower risk of colon cancer, but not rectal cancer, for highest *vs* lowest WG product intake. In the above-mentioned NIH-AARP Diet and Health study, there were also indications of lower risk of colorectal cancer for a higher WG intake, although the association was nonsignificant and somewhat weaker than that observed for men in the same cohort (Schatzkin *et al*, 2007).

It is unclear why our study suggests an association for men but not for women. Possible reasons for the apparent lack of associations between intake of WG products and risk of colon and rectal cancer among women in our study may concern issues regarding lack of sufficient variation in intake of WG products among our female cohort participants to produce an effect, and/or lack of sufficient statistical power in the female-specific analyses, as fewer colon and rectal cancer cases were diagnosed in women than among men. In addition, based on our and the results obtained from the NIN-AARP Diet and Health study, it could be speculated whether the effect of WG products is stronger for men than it is for women. Suggestions of sex-specific effects of other diet-related factors with the development of colon and rectal cancer have been indicated previously (Jacobs, 2007) and what initially motivated us to generally conduct sex-specific analyses. Nonetheless, the gender-specific differences in associations observed in this study require further confirmation.

Small experimental and human intervention studies suggest that in particular WG rye products may hold potential positive effects in relation to colon and rectal cancer prevention that are superior to those of other WG cereals (e.g., WG wheat- and oat-based products; McIntosh *et al*, 2003; Kallio *et al*, 2008; Andersson *et al*, 2010). However, epidemiological data directly examining the effects of different WG products made from different cereal grains (e.g., WG rye, WG wheat and oat) on the risk of colon and rectal cancer are sparse, making comparison of our results with previous studies limited. With regard to specific types of WG products, a lower risk of colon cancer among men was most evident for WG bread intake, although a trend towards lower risk of colon cancer with higher consumption of WG rye bread was also apparent. Although this finding was of smaller magnitude in comparison to that of WG bread, it agrees with results from a Finnish cohort study in which a preventive role of rye foods in relation to risk of colorectal cancer in men was also suggested. Although in our study we reported no beneficial effects of WG rye bread intake in women, a significant protective effect of rye bread on risk of colon cancer was observed among women in the Swedish Mammography Cohort (Larsson *et al*, 2005). Within our cohort it is assumed that the WG bread consumed by our cohort participants is mainly WG wheat based, and thus our data do not strongly indicate differential effects of WG rye- wheat-based products, but this needs confirmation by other studies. In contrast, we did not find that oatmeal intake was associated with the risk of colon or rectal cancer; however, we cannot rule out whether the limited range of intake of oatmeal in our study could have contributed to the null finding for this specific WG product. Future studies should attempt to examine the associations of risk with specific types of WG products.

If colon and rectal cancers are aetiologically distinct (Potter, 1996; Wei *et al*, 2004), proximal and distal colon cancers can also be considered as different classes of colon cancer (Bufill, 1990; Iacopetta, 2002). Our results showed that higher total WG product consumption was associated with a significant lower risk of proximal colon cancer and an indication of a lower risk of distal colon cancer as well. Although one large case–control study reported no association with proximal or distal colon cancer risk (Hu *et al*, 2007), our findings are consistent with those of two other prospective studies showing suggestive protective effects of high WG consumption in these cancers (Larsson *et al*, 2005; Schatzkin *et al*, 2007). At this point there is no strong indication that a preventive effect of WG product may vary substantially according to specific colon subsite.

In conclusion, we found that a higher intake of total WG products is associated with a lower risk of colon and perhaps also rectal cancer in men, but not in women. Intake of WG bread in particular seemed to account for the association between total WG products and risk of colon cancer in men. Overall, these data, especially for men, provide further support to the public health message to increase the intake of WG products in gaining optimal health.

## Figures and Tables

**Table 1:** Baseline characteristics and wholegrain consumption among the total cohort and colon and rectal cancer cases presented separately for men and women, the Danish Diet, Cancer and Health study

	**Men**			**Women**		
	**Total cohort (*n*=26 630)**	**Colon cancer cases (*n*=244)**	**Rectal cancer cases (*n*=169)**	**Total cohort (*n*=29 189)**	**Colon cancer cases (*n*=217)**	**Rectal cancer cases (*n*=114)**
*Age at baseline, years*						
Median (5th–95th percentile)	56 (50–64)	59 (51–64)	58 (51–64)	56 (50–64)	58 (51–64)	58 (50–65)
						
BMI, kg m^–2^						
Median (5th–95th percentile)	26 (21–33)	26 (21–34)	27 (21–32)	25 (20–34)	26 (20–37)	26 (20–35)
						
*Alcohol intake, g per day*						
Median (5th–95th percentile)	20 (2–80)	25 (3–82)	22 (3–90)	10 (1–42)	8 (1–41)	6 (1–42)
						
*Red and processed meat intake, g per day*						
Median (5th–95th percentile)	139 (67–255)	142 (71–260)	140 (58–249)	84 (35–157)	86 (35–154)	84 (26–154)
						
*School education, n* (%)						
Low (⩽7years)	9242 (35)	83 (34)	54 (32)	9141 (31)	84 (39)	41 (36)
Medium (8–10 years)	11 070 (42)	111 (45)	82 (49)	14 672 (50)	98 (45)	55 (48)
High (⩾11years)	6318 (24)	50 (20)	33 (20)	5376 (18)	35 (16)	18 (16)
						
*Physical activity, a total no. of activities* a						
Median (5th–95th percentile)	5 (2–5)	4 (2–5)	5 (2–5)	4 (2–5)	4 (2–5)	4 (2–5)
						
*HRT status, n* (%)						
Never user	—	—	—	15 901 (54)	131(60)	68 (60)
Past user	—	—	—	4522 (15)	30 (14)	16 (14)
Current user	—	—	—	8766 (30)	56 (26)	30 (26)
						
*Total wholegrain products, g per day*						
Median (5th–95th percentile)	130 (42–267)	115 (37–243)	118 (55–241)	113 (31–224)	113 (38–220)	103 (28–282)
						
*Wholegrain Rye bread, g per day*						
Median (5th–95th percentile)	63 (20–163)	63 (20–163)	63 (20–163)	63 (11–113)	63 (11–113)	63 (20–113)
						
*Wholegrain bread, g per day*						
Median (5th–95th percentile)	31 (1–100)	17 (1–100)	31 (1–100)	31 (1–100)	31 (1–100)	31 (1–180)
						
*Oatmeal, g per day*						
Median (5th–95th percentile)	21 (1–50)	21 (1–50)	4 (1–50)	7 (1–50)	7 (1–50)	4 (1–50)

Abbreviations: BMI=body mass index; HRT=hormone replacement therapy.

a The median refers to the summarised number of the leisure time physical activities, that is, walking, cycling, sports, gardening, housework and do-it-yourself work. Participation is defined as >0 h of activity.

**Table 2:** Incidence rate ratios (IRR) and 95% CIs for colon and rectal cancer in men and women according to intake of total and individual wholegrain products, the Danish Diet, Cancer and Health study

	**Colon cancer**			**Rectal cancer**		
**Wholegrain product variables**	**No. of cases**	**Crude IRR (95% CI)** a	**Adjusted IRR (95% CI)** b	**No. of cases**	**Crude IRR (95% CI)** a	**Adjusted IRR (95% CI)** c
*Men*						
Total wholegrain products (per every 50 g increment) By categories of daily total wholegrain product intake (g per day)	244	0.83 (0.76–0.92)	0.85 (0.77–0.94)	169	0.88 (0.79–0.98)	0.90 (0.80–1.01)
Group 1: ⩽75 (g per day)	61	1.00	1.00	32	1.00	1.00
Group 2: 75 to ⩽115 (g per day)	61	0.91 (0.64–1.30)	0.95 (0.66–1.35)	49	1.40 (0.90–2.19)	1.45 (0.93–2.26)
Group 3: 115 to ⩽160 (g per day)	46	0.66 (0.45–0.96)	0.70 (0.47–1.03)	30	0.81 (0.49–1.33)	0.84 (0.51–1.39)
Group 4: >160 (g per day)	76	0.56 (0.40–0.78)	0.61 (0.43–0.86)	58	0.82 (0.53–1.26)	0.88 (0.57–1.36)
Wholegrain Rye bread (per every 25 g increment)^d^	244	0.93 (0.87–1.00)	0.94 (0.88–1.01)	169	0.96 (0.88–1.04)	0.97 (0.89–1.05)
Wholegrain bread (per every 25 g increment)^d^	244	0.89 (0.82–0.96)	0.89 (0.82–0.97)	169	0.94 (0.86–1.03)	0.95 (0.86–1.04)
Oatmeal (per every 25 g increment)^d^	244	0.94 (0.77–1.15)	0.95 (0.77–1.17)	169	0.79 (0.61–1.03)	0.80 (0.62–1.04)
						
*Women*						
Total wholegrain products (per every 50 g increment) By categories of daily total wholegrain product intake (g per day)	217	0.96 (0.86–1.07)	0.98 (0.88–1.10)	114	1.01 (0.87–1.18)	1.02 (0.88–1.19)
Group 1: ⩽75 (g per day)	57	1.00	1.00	38	1.00	1.00
Group 2: 75 to ⩽115 (g per day)	62	1.03 (0.72–1.47)	1.08 (0.75–1.56)	28	0.69 (0.43–1.13)	0.70 (0.43–1.14)
Group 3: 115 to ⩽160 (g per day)	47	1.05 (0.71–1.54)	1.13 (0.77–1.67)	16	0.54 (0.30–0.96)	0.54 (0.30–0.97)
Group 4: >160 (g per day)	51	0.85 (0.58–1.23)	0.92 (0.63–1.35)	32	0.80 (0.50–1.28)	0.81 (0.50–1.30)
Wholegrain Rye bread (per every 25 g increment)^d^	217	1.01 (0.92–1.10)	1.01 (0.92–1.11)	114	1.04 (0.92–1.17)	1.04 (0.92–1.18)
Wholegrain bread (per every 25 g increment)^d^	217	0.96 (0.89–1.05)	0.97 (0.89–1.06)	114	1.04 (0.94–1.16)	1.05 (0.94–1.16)
Oatmeal (per every 25 g increment)^d^	217	1.12 (0.91–1.38)	1.14 (0.92–1.40)	114	0.80 (0.58–1.11)	0.81 (0.58–1.12)

Abbreviations: IRR=incidence rate ratio; CI=confidence interval.

a Unadjusted analysis.

b Analysis adjusted for body mass index, alcohol intake, school education, intake of red and processed meat, use of hormone replacement therapy (women only) and leisure time physical activity.

c Analysis adjusted for body mass index, alcohol intake, school education and intake of red and processed meat and use of hormone replacement therapy (women only).

d The variables were mutually adjusted in analyses.
